# Claudins: from gatekeepers of epithelial integrity to potential targets in hepato-pancreato-biliary cancers

**DOI:** 10.3389/fonc.2024.1454882

**Published:** 2024-09-26

**Authors:** Hyein Jeon, Michelle Sterpi, Christiana Mo, Fernand Bteich

**Affiliations:** ^1^ Department of Medical Oncology, Albert Einstein College of Medicine, Bronx, NY, United States; ^2^ Department of Medical Oncology, Montefiore Medical Center, Bronx, NY, United States

**Keywords:** claudins, hepatocellular carcinoma, gallbladder cancer, bile duct cancer, pancreatic cancer, cancer therapeutics, targeted therapy

## Abstract

Claudins, a family of tetraspan transmembrane proteins, are critical to the integrity of tight junctions in epithelia and endothelia, influencing cellular processes such as development, differentiation, and apoptosis. Abnormal claudin expression is associated with various malignancies, particularly affecting tissue architecture and potentially facilitating tumor invasion and metastasis. In this comprehensive review, we explore the multifaceted functions of claudins: their expression, specific roles in cancer with a focus on hepato-pancreato-biliary malignancies and highlight their potential as therapeutic targets. We discuss current claudin-targeted therapies, including monoclonal antibodies, antibody-drug conjugates, bispecific T-cell engager and chimeric antigen receptor T-cell therapies. These approaches show promise in pre-clinical and clinical studies, particularly in hepato-pancreato-biliary cancers with large unmet needs. Despite these early signs of efficacy, challenges remain in effectively targeting these proteins due to their structural resemblance and overlapping functions.

## 1 Introduction

Claudins (CLDN) represent a family of transmembrane proteins that are fundamental constituents of tight junction (TJ) strands between both epithelial and endothelial cells. These proteins, pivotal for maintaining intercellular barrier integrity, are also involved in regulating selectivity for ions and small molecules transport across the paracellular space. CLDNs possess the structural hallmark of tetraspanin proteins, featuring four transmembrane helices (1). To date, there are 27 known members of the CLDN family(2). The heterogeneity of CLDN expression in tissues and their interactive roles within the TJ complex have illuminated intriguing avenues of research, leading to a deeper understanding of physiological processes, pathological conditions, and potential therapeutic interventions centered on them.

CLDNs are categorized into “classic” and “non-classic” groups based on their structural differences (1). In addition to their ubiquitous barrier and selectivity function, CLDNs play significant roles in various cellular processes including development, differentiation, and cellular apoptosis, primarily through their interactions with cytoplasmic proteins and their participation in signal transduction pathways (1, 3, 4). Abnormal expression or function of CLDNs is commonly observed in malignancies, where changes in their spatial distribution can disrupt tissue architecture and barrier functions, potentially facilitating tumor invasion and metastasis, as well as transduction of signals that are important in tumorigenesis (5, 6). These cancer-associated changes have made CLDNs a focus of research for understanding tumor biology and for exploring these proteins as viable drug targets.

This extensive review delves into the diverse functions of CLDNs in cancer, emphasizing their potential as therapeutic targets and highlighting ongoing claudin-directed research efforts. We particularly focus on the specific roles of CLDNs in hepato-pancreato- biliary cancers, given the urgent need for novel treatment approaches in this area.

## 2 Claudin structure and function

**Figure 1:**
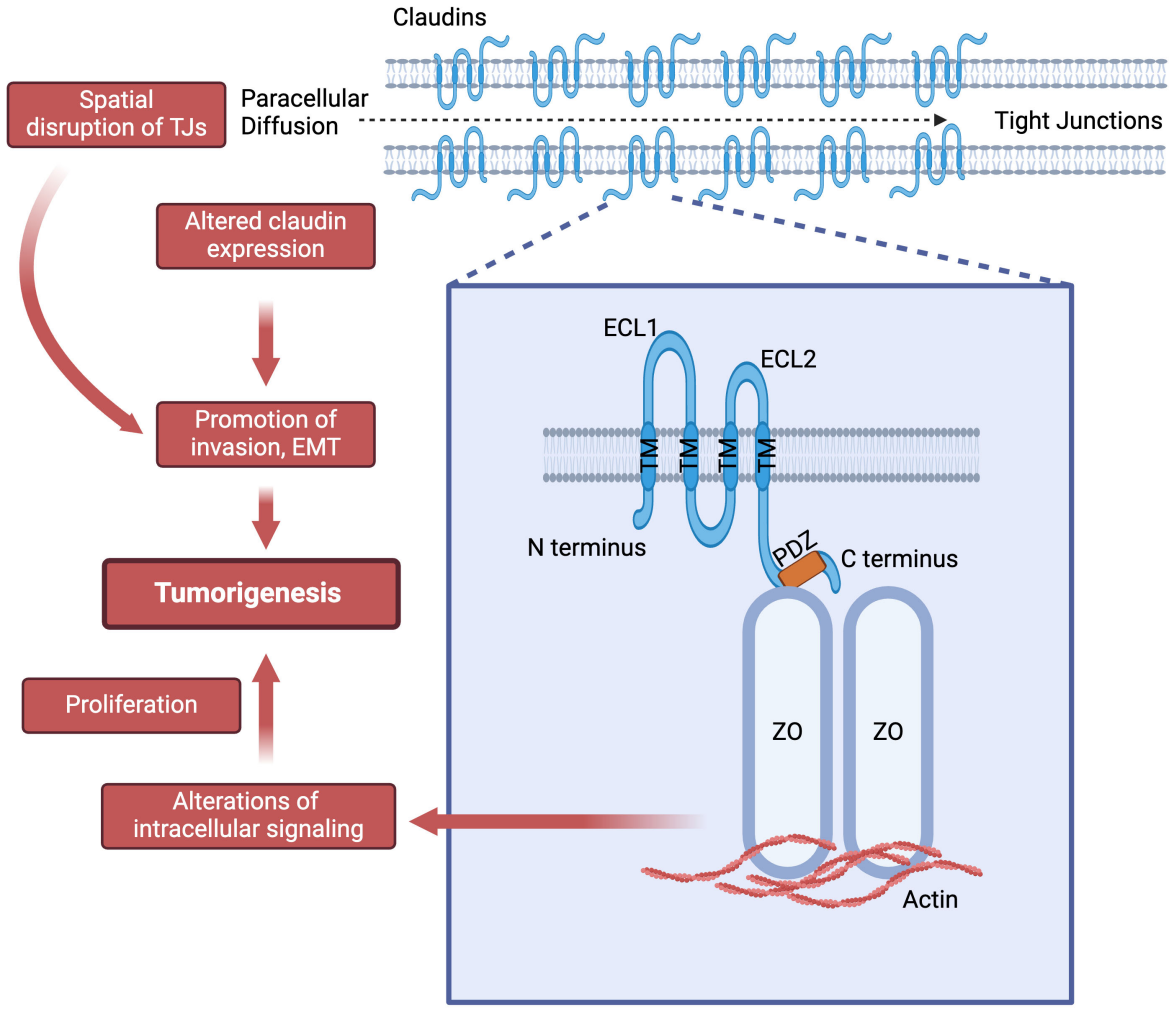
Claudins structure and their potential role in tumorigenesis. ECL: extra-cellular loop; PDZ: Post synaptic density protein-95, disc large and zonula occludens-1 (PDZ) domain binding motif; TM: transmembrane helices; ZO: Zonula occludens-1.

CLDNs are integral membrane proteins found in TJs, with a molecular weight of approximately 20-27 kDa. They possess four transmembrane domains and two extracellular loops that contribute to the structural integrity and selective permeability of TJs (**Figure 1**). CLDNs polymerize linearly at the apical portion of lateral plasma membranes of cells to create a foundation for TJ and function as paracellular barrier (7). Due to its larger size, the first extracellular loop plays a crucial role in the barrier and selective pore-forming properties of CLDNs, dictating their specific ion selectivity and contributing significantly to the mechanical strength of TJ (7).

The N- and C-termini are located intracellularly, where they engage in interactions with various cytoplasmic proteins such as the zonula occludens proteins at the PDZ domain binding motif and partake in intracellular signal transduction events. CLDNs are subdivided into two groups: classic (1-10, 14,15, 17, 19) and non-classic (11-13, 16, 18, 20-27) based on sequence analysis similarities (1). This classification is based on the presence or absence of two extracellular loops in these proteins (1, 8).

CLDNs facilitate interactions with signaling proteins such as Src-family kinases (SFKs) through their second extracellular loop (ECL2) and C-terminal intracellular domain, thereby activating the PI3K/AKT/SGK pathway (3, 5, 9). This activation subsequently triggers transcription factors like Retinoid X Receptor α (RXRα)/Retinoic Acid Receptor γ (RARγ) or estrogen receptor α (ERα), which regulate genes crucial for cellular proliferation, apoptosis and homeostasis (10).

## 3 Claudin alterations in malignancies

Aberrant expression or dysregulation of the function of CLDN are frequently observed in various malignancies (11–14). These changes can compromise tissue architecture, disrupt the natural barrier function of CLDN, alter cell polarity and potentially pave the way for tumor invasion and metastasis. Altered expression of CLDN in tumors, elucidated through immunohistochemical techniques, has also been predominantly associated with poor prognosis in numerous studies, highlighting its critical role as a prognostic marker in patient assessment (15–17).

CLDN18.2, a splice variant of CLDN 18 (see section 4.8 for further discussion) and CLDN6 are the main target of therapeutic approaches due to their absent or near absent expression in normal organs such as lung, or liver (18–21). Expression of CLDN18.2 in normal pancreatic tissue is near absent while it is upregulated in pancreatic tumors (18, 19). Conversely, gastric mucosa seems to selectively express and retain CLDN18.2 expression in normal and malignant cells while its expression was absent in gastric stem cells (19, 20). CLDN6, which has tight suppression in normal human epithelial and endothelial cells, is commonly expressed in germline tumors such as testicular and ovarian cancers (21).

Although extensive research has characterized CLDN expression in cancer, accurately delineating cancerous tissue-specific expression and understanding the diverse roles of different CLDN types in cancer continue to pose significant challenges. Furthermore, the functions of various CLDNs, particularly in hepatobiliary and pancreatic cancers, are still being explored and can often appear contradictory between cancer types. For instance, expression of certain CLDNs is sometimes decreased in carcinogenesis (15, 16, 22). In **Table 1**, however, we list many CLDNs that are overexpressed in hepato-pancreato-biliary cells – mainly with altered localization-, suggesting their potentials for targeted novel therapies (40). Additionally, CLDN expression can exhibit heterogeneity within a single tumor type, adding to the complexity of this tightly regulated protein in tumor biology (41). Given the high unmet need in hepatobiliary and pancreatic cancer (42), the potential of CLDNs as therapeutic targets necessitates further exploration, underscoring the critical need for continued research in this area.

**Table 1:** Overview of Claudin.

Claudin	Normal Functions	Tumor Type	Signaling Pathway	Claudin Expression in Tumor vs. Normal	Primary Mechanism in Tumorigenesis	Citations
CLDN 1	Involved in epithelial barrier functions Ubiquitously expressed in different organs	Pancreatic	PKC/MAPK/ERK pathway TNF- α/TGF-β1 pathway	↑/↓	Decreased cell-cell adhesion during EMT Promotes tumor invasion and metastasis	(23, 24)
		HCC	c-Abl-PKCδ pathway ZEB1 and Slug transcriptional factors	↑/↓	Increases MMP2 which leads to cell invasion, and migration	(25, 26)
CLDN 3	Maintains the blood-biliary barrier Expressed in most organs	HCC	Wnt/β-catenin pathway	↓	Promotes cell migration and invasion	(22)
CLDN 4	Involved in epithelial differentiation, polarity maintenance, and trafficking Receptor for *C. perfringens enterotoxin* (*CPE*)	Pancreatic	PI3K/AKT/ERK1/2 signaling YAP1 activation	↓	Promotes tumor formation, invasion, and metastasis	(27, 28)
		HCC	Wnt/β-catenin pathway	↓	Promotes tumor formation, invasion, and metastasis	(29)
CLDN 6	Expressed in embryonic stem cells Minimal expression in normal adult organs	Gastric/GEJ	YAP1/LATS1/2 pathway Activates MMP2	↑	Promotes invasion and metastasis	(30)
		HCC	Receptor for HCV EGFR/akt/mTOR pathway	↑	Promotes invasion and metastasis	(30)
CLDN 7	Impermeable claudin	Pancreatic	ERK1/2 pathway EpCAM-CLDN-7 complex	↑	Promotes tumor formation and accelerates tumor growth	(31)
		HCC	ERK1/2 pathway	↑	Promotes metastasis	(32)
CLDN 10	Involved in paracellular sodium permeability	HCC	MMP and MT1-MMP pathway	↑	Promotes intrahepatic metastasis	(33)
CLDN 17	Forms anion-selective channels	HCC	Tyk2/Stat3 pathway	↑	Promotes tumor formation, invasion, and metastasis	(34)
CLDN 18.2	Found in the epithelium of normal gastric mucosa Controls paracellular H+ transport	Gastric/GEJ	PKC/MAP/AP-1 pathway	↑/↓	Promotes tumor formation, invasion, and metastasis	(35–37)
		Pancreatic	PKC/MAP/AP-1 pathway EGFR pathway	↑	Promotes tumor formation, invasion, and metastasis	(18)
		Biliary Tract	PKC/MAP/AP-1 pathway ERK1/2 pathway	↑	Increases the invasiveness of malignant bile duct cells	(38)
CLDN 23	Participates in signaling pathways and cell differentiation	Pancreatic	MEK/ERK pathway	↓	Potentiates tumor invasion and metastasis	(39)

PKC, protein kinase C; MAPK, mitogen-activated protein kinase; ERK, extracellular regulated kinases; TNF, tumor necrosis factor; TGF, tumor growth factor; P13K, phosphoinositide 3-kinase; AKT, protein kinase B; YAP, Yes-associated protein; LATS, large tumor suppressor; MMP, matrix metalloproteinase; EpCAM, epithelial cellular adhesion molecule, EGFR, epidermal growth factor receptor; mTOR, mammalian target of rapamycin; MT, membrane type; MEK, MAPK/ERK kinase. ↑, Increased expression in tumor vs. normal; ↓, decreased expression in tumor vs. normal.

## 4 Claudins in hepato-pancreato-biliary malignancies: expression and roles

In the subsequent sections, we will explore the roles of CLDNs 1, 3, 4, 6, 7, 10, 17, 18.2, and 23 in hepatobiliary and pancreatic cancers (PC). We have selected the above listed CLDNs, taking into account their significant roles in the pathogenesis of the hepato-pancreato-biliary cancers (**Table 1**) and the promising data suggesting their therapeutic potential (35, 37, 43). The CLDNs will be presented sequentially, starting with CLDN1 and concluding with CLDN23.

### 4.1 Claudin 1

CLDN1, the first identified member of the CLDN family integral to TJs, is a 22kDa membrane protein expressed in various organs such as the intestine, spleen, brain, liver, kidney, and testis (4, 25, 26, 44) among others^1^. While it plays a role within TJs, CLDN1 is also expressed outside of these protein complexes. Non-junctional CLDN1 (NJ-CLDN1), for example, is found in the basolateral membrane of hepatocytes (45) and facilitates the entry of hepatitis C virus (HCV) through the sinusoidal blood (23). Additionally, mis-localization of CLDN1 from the cell membrane to the cell nucleus and cytoplasm, as observed in human tumor samples (46), suggests its oncogenic potential.

The role of CLDN1 in hepatocellular carcinoma (HCC) is crucial but complex. While we speculate CLDN1 might have an oncogenic role in HCC, there is no definitive proof of a direct causal relationship between CLDN1 and oncogenesis. Significantly increased expression of CLDN1 was found in precancerous dysplastic nodules of cirrhosis and in HCC, especially in high grade tumors (47). Data retrieved from the Genomic Data Commons Data Portal and the human protein atlas showed that CLDN1 is the most highly expressed member of the CLDN family at both the mRNA and protein levels in HCC (48, 49).

One hypothesis is driven by the disruption of linear distribution and localization of CLDN1, especially in HCV mediated HCC, through down-regulation or silencing of *tacstd2* and Trop2 (50, 51). Localization of CLDN1 can be aberrantly non-junctional in HCC (47) and this may be regulated by the tumor-associated calcium signal transducer 2 (*tacstd2*), which encodes the transmembrane glycoprotein Trop2 (52). Trop2 is commonly overexpressed in various carcinomas associated with tumor cell plasticity, growth and metastasis and CLDN1 with CLDN7 acts as a ligand for Trop2 (52), suggestive of its potential role in tumorigenesis via CLDN1 mediated manner.

Several other hypotheses remain regarding how CLDN1 might contribute to HCC formation and metastasis. C/D box 126 (SNORD126), a small nucleolar RNA (snoRNA) associated with HCC, activates the PI3K-AKT pathway, which plays an important role in tumorigenesis and increases CLDN1 expression (53). CLDN1 could also be disrupted due to changes in EMT via several modulatory molecules, such as tumor necrosis factor α (TNF-α)/NF-κB, human growth hormone, and the receptor for glial cell-line derived neurotrophic factor (GFRα1) affecting HCC cell proliferation, survival, migration and invasion (47). The supposed role of CLDN1 as an oncogene and EMT promoter might be mediated by the c-Abl-protein kinase C delta (PKC)δ signaling pathway and the activation of metaloproteinase-2 (MMP2) (25, 26). It is worth noting however that there are studies that correlate lower CLDN1 expression in HCC with dedifferentiation, portal invasion, and reduced post-hepatectomy survival rates (54), and CLDN1 overexpression with inhibition of EMT in the human cell line Huh7 (55, 56). These conflicting findings ultimately suggest that our under-standing of the relationship between CLDN1, EMT and tumorigenesis currently is still limited and warrants further investigations.

The recent use of humanized monoclonal antibodies (mAb) targeting NJ-CLDN1 detected predominantly non-junctional up-regulation of CLDN1 in HCC, as opposed to its mainly junctional placement in healthy liver tissue (47). Targeting NJ-CLDN1 significantly suppressed tumor growth and invasion in HCC models, suggesting its role in tumor progression, plasticity, and signaling. Furthermore, CLDN1 overexpression on tumor cells was found to contribute to an immunosuppressive tumor microenvironment (TME) and may influence responses to immune checkpoint inhibitors (47).

In another setting, there is increased expression of CLDN1 in pancreatic duct adenocarcinomas (PDAC) and intraductal papillary mucinous pancreatic tumors (IPMN) (14, 44). Immunohistochemical (IHC) analyses showed CLDN1 overexpression in 58% of PDAC and 20% of invasive IPMN lesions (14). Its expression in PC is also aberrant, with errors in the nuclear-cytoplasmic membrane localization observed in metastatic tissues (28).

Exploring the impact of tumor necrosis factor-alpha (TNF-α) and transforming growth factor-beta 1 (TGF-β1) on CLDN1 expression sheds light on its effects on tumorigenesis (57). Treatment of PANC-1 pancreatic cancer cells with TNF-α has revealed that CLDN1 expression may mediate TNF-α-induced signals, leading to apoptosis and inhibition of cell proliferation (45). Similarly, inhibition of PKCα with Gö6976 has been shown to prevent TGF-β1-induced epithelial-mesenchymal transition (EMT) and subsequent downregulation of CLDN1 in PC cells, suggesting that PKCα influences CLDN1 downregulation through the MAPK/ERK pathway and Snail regulation in PC cells (24, 57, 58). Moreover, studies utilizing a Panc-1 xenograft model in mice have investigated the impact of miR-193b on CLDN1 and its effects on PC. Decreased miR-193b levels in PC cells potentially contribute to tumor progression, while introducing miR-193b demonstrates promise in inhibiting cell proliferation, migration, invasion, and EMT. These effects, consistent with the aforementioned findings, are ascribed to the increased CLDN1 expression, along with the downregulation of Snail and TCF8/ZEB1, driven by the eEF2K and MAPK/ERK pathways (59). Interestingly, a novel EMT-inhibiting compound called C150 demonstrates promise in reducing PC growth and metastasis. *In vitro* experiments show that C150 significantly increases CLDN1 expression, while *in vivo* studies indicate a non-significant trend towards its elevation (23).

The role of CLDN1 in biliary cancers, such as gallbladder cancer (GBC) and biliary tract cancers (BTC), is not fully understood. Available data suggest its involvement in tumor invasion and metastasis. However, there are contrasting findings regarding the relationship between CLDN1 regulation and cancer cell proliferation . Xiong et al in 2011 (60) observed lower expression of CLDN1 in surgically resected GBC tissue specimens compared to peritumoral tissues, adenomatous polyp, or chronic cholecystitis (60). In the same study, higher CLDN1 expression was associated with lower tumor grade and better prognosis. This finding is in line with the hypothesis that the loss of intercellular adhesion may breach the cellular junctions and is associated with tumor invasion and metastasis (14). A later investigation conducted in 2019 on GBC and cholangiocarcinoma (CCA) cell lines indicated that downregulation of CLDN1 might promote apoptosis and inhibit cell invasion, potentially contributing to recurrence and metastasis in GBC (28).

Additionally, IHC analysis of normal and neoplastic biliary tract tissues revealed that CLDN1 expression in extrahepatic BTC and GBC seems to inversely correlate with increasing tumor grade (61). Interestingly, in the same study intrahepatic BTC showed significantly higher CLDN1 expression compared to extrahepatic BTC and GBC. These differential expression patterns, along with other CLDNs discussed in the review, could potentially aid in differentiating and understanding the biological behavior of these cancers.

### 4.2 Claudin 3

CLDN3 is a widespread protein that has known associations with progression in multiple malignancies including glioblastoma multiforme (62) and gastric cancer (63). In other contexts, it exhibits a contrasting effect, acting as a suppressor of the Wnt/β-catenin signaling pathway, thereby hindering EMT and reducing the malignant potential of cells in HCC, colorectal cancer, and lung squamous cell carcinoma (22, 64, 65). CLDN3 can be found in normal hepatocytes and biliary tract cells, and primarily forms TJs that maintain the blood-biliary barrier. In mice models, decreased CLDN3 led to a weakened barrier, lowered lipid metabolism, and slower hepatocyte regeneration (66). Conversely, increased CLDN3 expression in malignant hepatocytes was associated with decreased cell migration and invasiveness (65). These observations, coupled with the decreased CLDN3 expression in HCC cells, all suggest that CLND3 has a tumor-suppressive function in HCC.

While the role of CLDN3 in biliary tract cancers is not fully understood, it holds promise as a potential diagnostic biomarker. In fact, CLDN3 can be identified in extracellular vesicles (EVs), which are secreted from cells. EVs can contain DNA, RNA, and proteins from their cells of origin (67). In bile-derived EVs, CLDN3 expression levels were significantly higher in cholangiocarcinoma-derived EVs compared to those from normal bile duct epithelium (68).

### 4.3 Claudin 4

CLDN4 is a promising target for antitumor drugs due to its dual role in cell adhesion in epithelial tumors and as a receptor for the C. perfringens enterotoxin (*CPE*) (69). A recent focus on uncovering a structure-based approach to hinder CPE cytotoxicity has enhanced our comprehension of the interplay between CPE and CLDN4 (70). In this suggested mechanism, CLDNs function not only as CPE receptors but also as integral components in the CPE cytotoxic process, where claudin-to-claudin interactions drive CPE oligomerization and facilitate crucial structural changes for β-pore formation (70).

The role of CLDN4 in cancer is even more intricate, as its increased expression can both promote and inhibit tumor development (28). In CLD4-overexpressing epithelial malignancies, it may promote a malignant cancer phenotype by maintaining the TME through the barrier function of TJs. Additionally, it can stimulate angiogenesis through VEGF and IL8 and contribute to an acidic environment that facilitates increased expression of malic enzyme 1 and EMT through YAP1 activation, while also suppressing immune cells. Thus, the repression of CLDN4 may suppress invasion and metastasis, as it has been observed in breast cancer cell lines (57).

On the contrary, accumulating knowledge indicates that CLDN4 overexpression may impede pro-oncogenic signaling from EphA2 (71). More specifically, by repressing the PI3K-AKT and β-catenin pathways, increased levels of CLDN4 might downregulate E-catenin and PI3K activity, thereby preventing AKT-mediated phosphorylation of EphA2 at S897 and potentially impeding cancer progression (71).

Of interest, beyond its role in TJs, CLDN4 has non-TJ functions that contribute to carcinogenesis and stemness through FAK signaling via integrin-β activation, YAP induction, and PI3K/AKT/ERK signaling (27, 72, 73).

Studies employing expression profiling and IHC analysis have shown that CLDN4 is highly expressed in both primary and metastatic PC tissue samples (74–76) and in precancerous conditions such as IPMN (14), pancreatic intraepithelial neoplasia (77) and mucinous cystic neoplasms (78). In PC, CLDN4 appears to be a potent inhibitor of the aggressive and metastatic phenotype of PC cells and its increased expression is associated with extended survival and reduced pulmonary metastases (74, 79). CLDN4 expression was also noted to be modulated by the Ras/Raf/ERK pathway: inhibition of both major downstream effectors of Ras, MEK and PI3K, was associated with decreased CLDN4 expression in PANC-1 cells. While on one hand transcriptional up-regulation of CLDN4 by Ras-activation could account for the observed high expression of CLDN4 in the majority of PC, on the other, it points to a complex regulation of the TJ constituents, with an unclear role of the effect of Ras-activation and its downstream effectors on CLDN expression (74, 80). Moreover, although a direct causal link between the downregulation of CLDN4 and tumor cell activities such as proliferation, migration, invasion, and tumorigenesis has not been definitively established, studies do suggest that the proinvasive TGF-β can reduce CLDN4 expression in PANC-1 cells and that low expression of CLDN4 is significantly associated with shorter survival in patients with PDAC (81, 82). This might be due to the reduced CLDN4 expression in endothelial cells, which in turn might decrease paracellular resistance and promote invasion of the epithelial cancer cells through the endothelial cell layer, similarly as to what was described for E-cadherin (28, 83).

Given the findings regarding the increased expression of CLND4 in PC, further comprehensive investigations are warranted to validate its role and potential implications in the development and progression of this cancer type. For example, the upregulation of CLDN4 expression induced by Troglitazone, a PPAR agonist, could potentially hinder cell growth, possibly through the interaction between CLDN and actin, ultimately resulting in suppressed cell motility and altered cell morphology (84–86). Therapeutic targeting of CLDN4 in PC also shows promise using specific mAbs that recognize its ECL2 domain. The mAb KM3934 demonstrated efficacy in inducing cytotoxicity in PC cells *in vitro* (87). These CLDN4-targeting mAbs, including 4D3, seem to have the potential to enhance chemotherapy effectiveness through synergistic inhibition of tumor growth (88).

Moreover, CLDN4 interactions are being investigated for disrupting the TME (89), improving drug delivery, and exploring targeted delivery and imaging methods for early detection and diagnosis of PC (90, 91). Promising results have been observed with radiolabeled mAbs (92) and fluorochrome-labeled C-CPE tracers in detecting PC and precursor lesions (93). CLDN4 was employed also as a target in a novel multifaceted detection platform utilizing a carrier modified with apoferritin, enabling efficient tumor targeting. However, further research is required to validate and optimize these approaches.

In the liver, under physiological conditions, CLDN4 is expressed in hepatic progenitor cells and bile duct epithelia, but not in mature hepatocytes (94, 95). In the cirrhotic liver tissue, CLDN4 expressions is found in hepatocytes in the peripheral region of regenerative nodules and correlates with the degree of fibrosis (79). In HCC specimens, the expression of CLDN4 is higher than in non-tumorigenic liver tissue, which could be due to cytokine storms that influence the gene expression of the hepatocytes. Decreased CLDN4 expression appears to be associated with high-grade tumors and tumor dedifferentiation, and may serve as an independent prognostic factor for worse survival prognosis and higher recurrence rates in HCC (29).

Despite the aberrant CLDN4 expression noted within the malignant hepatocytes, CLDN4 is undetectable in the majority of HCC cases and it may therefore prove useful to help distinguish between HCC and BTCs, as it exhibits strong expression in BTCs (96–98). Specifically, elevated CLDN4 levels in CCA cell lines have been correlated with tumor migration and invasion, indicating a potential role of CLDN4 in promoting expansion and metastasis in this type of BTC (11). Therefore, CLDN4 expression can further discriminate between extrahepatic and intrahepatic CCAs due to its heightened immunoreactivity in the former as compared to the latter. It has also been suggested as a useful marker to distinguish between intrahepatic CCAs and HCC or metastatic adenocarcinoma (61).

### 4.4 Claudin 6

Located on chromosome 16p13.3, CLDN6 has a molecular weight of 23 kDa. Its C-terminal in the cytoplasm binds to the PDZ proteins for regulation of signal transduction, cell polarity, and synaptic transmission (8).

CLDN6 can form homotypic interactions among its CLDNs, as well as heterotypic interactions with other TJ proteins, thereby facilitating a network of multitudes of adhesion combinations. These interactions are crucial for the assembly and function of TJ. CLDN6 expression is most prominent in the early embryonic stages but diminishes in most tissues after birth (21), suggesting its role in fetal development, cell differentiation, and tissue morphogenesis (8).

It has been noted that malignancies in various organ types aberrantly express CLDN6 (8, 21, 99–102), while its presence in normal adult tissues is tightly suppressed (21, 100). In the case for gastrointestinal and hepatobiliary malignancies, high levels have been noted in gastric adenocarcinoma (17, 30, 102), biliary tract (21), PC (21) and HCC (103, 104) with poor prognosis.

Previous studies have shown several regulation mechanisms associated with CLDN6. In gastric cancer, CLDN6 promotes tumor growth via the YAP1-LATS1/2 pathway, affecting the endothelial-mesenchymal transition process in tumor metastatic ability (30). In contrast to CLDN1, CLDN6 activates metaloproteinase-2 (MMP2), another mechanism to facilitate invasion and tumor migration (105). In breast cancer, high expression of CLDN6 showed resistance to doxorubicin, 5-fluorouracil, and cisplatin, while suppression of CLDN6 showed enhanced killing effects. This mechanism is thought to involve glutathione S-transferase-1 through the inhibition of p53 translocation to the cytoplasm, leading to drug resistance (106). This finding could have potential implications for patients who are refractory to the aforementioned chemotherapies.

CLDN6 is highly expressed in HCC and also acts as a receptor for HCV. It facilitates virus entry and replication, working in conjunction with CLDN-1 and CLDN-9 (104). The upregulation of CLDN6 augments the EMT phenomenon, enabling further invasion and metastasis through the EGFR/AKT/mTOR pathway. In contrast, decreased expression of CLDN6 inhibits the metastatic potential and growth of HCC (104).

### 4.5 Claudin 7

CLDN7, part of the large CLDN protein family, has shown intriguing but somewhat contradictory roles in liver, pancreatic, and biliary cancers. It is known to interact with epithelial cell adhesion molecule (EpCAM), strengthening epithelial cell junctions. In HCC, some studies suggest CLDN7 is overexpressed, particularly in cirrhosis-associated HCC, while others propose down-regulation of CLDN7 as an independent positive prognostic factor, possibly linked to better differentiated hepatic tumor cells (29, 107). In PC, CLDN7’s role remains elusive. *In vitro* studies suggest it may activate ERK1/2 signaling involved in cellular proliferation and differentiation, while other work points to a favorable prognostic factor (31, 32). Further research is needed to clarify CLND7’s precise role and therapeutic potential in cancer.

### 4.6 Claudin 10

The expression of CLDN10 has been examined in a select group of studies focusing on hepato-pancreato-biliary cancers. In HCC, increased expression of CLDN10 were associated with poor survival and increased recurrence rates, indicating a possible role as a molecular marker for disease relapse after a curative-intent hepatectomy (108). Although CLDN10’s role in tumorigenesis remains mostly unexplored, it might be related to the upregulation of MMP-2 and protein expression of membrane type 1 (MT1)-MMP (109), which were demonstrated to promote intrahepatic metastases in HCC.

Similarly to CLDN1, CLDN10’s expression was noted to be stronger in intrahepatic BTC than in both extrahepatic bile duct and gallbladder cancers. A study suggested that biliary tract cancers could be distinguished from each other by the relatively strong expression of one or more CLDNs, namely CLDN1 and CLDN10 in intrahepatic BTC, CLDN2 in GBC, and CLDN4 for extrahepatic BTC (61).

### 4.7 Claudin 17

CLDN17 is a paracellular channel-forming TJ protein that creates anion-selective channels (110). Limited data are available on CLDN17 in pancreato-biliary malignancies. Literature has shown that CLDN17 is found to be strongly expressed in HCC cells, but weakly expressed in non-neoplastic hepatocyte cell line (34). CLDN17 stimulates the Tyk2/STAT3 signaling pathway, leading to increased levels of STAT1, STAT3, and Tyk2. Activation of this pathway enhances the migration and invasion potential of cells. *In vitro* studies have demonstrated decreased invasiveness after silencing Tyk2 in hepatocytes that express CLDN17 (34). This suggests that elevated levels of CLDN17 is a driving force for the development and progression of HCC. Furthermore, patients with increased CLDN17 expression in HCC tumors had a shorter overall survival compared to those with negative CLDN17 expression. CLDN17 has also been linked to HCC occurrence, metastasis, higher histological grade, and TNM stage, which indicates its potential as a poor prognostic indicator (34).

### 4.8 Claudin 18.2

The human CLDN18 gene locus is found on chromosome 3q22 and contains 6 exons and 5 introns. It encodes for a classical CLDN composed of two extracellular loops, four transmembrane domains, and a cytoplasmic domain (37). CLDN18 has two alternative first exons, leading to two spliced variants, CLDN18.1 and CLDN18.2, with highly homologous amino acid sequences that differ in the N-terminal 69 amino acids (37, 111). CLDN18.1 is expressed in lung alveolar epithelium, functioning primarily to regulate solute and ion permeability. CLDN18.2 is found in the stomach and controls the transport of hydrogen ions to block gastric acid leakage (35). CLDN18.2 is located in the differentiated epithelial cells of the gastric mucosa, but not in the gastric stem cells. The expression of CLDN18.2 is retained after malignant transformation and is found in up to 70% of primary and metastatic gastric adenocarcinoma, including lymph nodes metastases (20, 112). There is also evidence of CLDN18.2 expression outside of gastric adenocarcinoma tissues, such as in pancreatic, biliary tract, ovarian, and lung tumors (37, 113).

CLDN18.2 has been extensively studied for its role in advanced gastric and gastroesophageal junction (GEJ) adenocarcinomas, leading to the development of new therapies (20, 114). In gastric cancer cells, CLDN18.2 is regulated by the methylation status of its promoter region and by the protein kinase C (PKC)/mitogen activated protein kinase (MAPK)/activator protein 1 (AP-1)-dependent pathway (35). The methylation of the CPG islands suppresses the binding of transcription factors to the promoter region of CLDN18.2, consequently leading to gene silencing (37). Additionally, AP-1 binds to the regulatory elements of the CLDN18.2 promoter to increase transcription. The PKC and MAPK/ERK pathways further upregulate CLDN18.2 transcription by stimulating the activation of AP-1 (115). These mechanisms and pathways are involved in tumor differentiation and migration, leading to tumorigenesis, proliferation, and metastasis. CLDN18.2 is normally inaccessible due to its location deep in the gastric mucosa. However, cell polarity is lost during malignant transformation, resulting in disruptions in the TJs which allow CLDN18.2 to migrate from its lateral position to the surface of the tumor cells (20). This exposes CLDN18.2 epitope to binding, proving it to be a novel valuable target in advanced gastric and gastroesophageal cancers.

Aside from gastric and gastroesophageal cancers, there is emerging evidence on the clinical significance of CLDN18.2 in pancreatic malignancies as well. In PC cells, CLDN18.2 is transcriptionally regulated by DNA methylation and PKC pathways. Pre-clinical studies demonstrated that both CLDN18.2 mRNA and CLDN18.2 protein were induced by the PKC activator 12-O-tertrdecanoylphorbol-12-acteate (TPA) in *in vitro* PC cell lines (12, 18). Conversely, a pan-PKC inhibitor blocked CLDN18.2 upregulation (12). Similar to gastric cancer cells, CLDN18.2 expression in PC cells is also regulated by DNA methylation of its promoter CpG islands (37). These signaling pathways are incredibly analogous to CLDN18.2 regulation in gastric cancer cells (115). Currently there are multiple ongoing clinical trials utilizing CLDN18.2-directed therapy for pancreatic malignancies (**Table 2**).

**Table 2:** Claudin 18.2-targeted Clinical Trial.

Trial Name	Phase	Country	Treatment Setting	Cancer type	Patient Population	Therapeutic agent	Treatment arms	Primary Endpoint
NCT03816163	II	International	1st line	Pancreatic	CLDN 18.2-positive, metastatic pancreatic adenocarcinoma	anti-CLDN 18.2 monoclonal antibody	Zolbexutimab + nab-paclitaxel + gemcitabine nab-paclitaxel + gemcitabine	DLT, OS
NCT04396821	I/II	US	1st line	Gastric/GEJ Pancreatic	CLDN 18.2-positive, untreated, unresectable or metastatic pancreatic adenocarcinoma	anti-CLDN 18.2 monoclonal antibody	TST001 + nab-paclitaxel + gemcitabine	DLT, MTD
NCT04400383	I	China	2nd/3rd line	Gastric Pancreatic	CLDN 18.2-positive locally advanced or metastatic solid tumors	anti-CLDN 18.2 monoclonal antibody	AB011 single agent, then AB011 + nab-paclitaxel + gemcitabine	DLT
NCT04581473	Ib/II	China	2nd/3rd line	Gastric/GEJ Pancreatic	CLDN 18.2-positive, advanced pancreatic cancer that failed 1 line of therapy	CAR-T	CT041 autologous CAR-T injection Paclitaxel, irinotecan, apatinib, or anti-PD-1 antibody	MTD, PFS
NCT05393986	I	China	2nd/3rd line	Gastric/GEJ Pancreatic	CLDN 18.2-positive, advanced pancreatic cancer that failed 1 line of therapy	CAR-T	CT041 autologous CAR-T injection	DLT, MTD
NCT05911217	Ib	China	Adjuvant	Pancreatic	CLDN 18.2- positive pancreatic adenocarcinoma after surgery and adjuvant chemotherapy	CAR-T	CT041 autologous CAR-T injection	DFS
NCT05539430	I	US	2nd/3rd line	Gastric/GEJ Pancreatic	CLDN 18.2-positive, unresectable or metastatic pancreatic adenocarcinoma	CAR-T	LB1908	RDE, RP2D
NCT04966143	I	China	2nd/3rd line	Pancreatic	CLDN 18.2-positive, recurrent or refractory pancreatic adenocarcinoma	CAR-T	LY011 injection	ORR
NCT04805307	I	China	2nd/3rd line	Gastric/GEJ Pancreatic	CLDN 18.2-positive, recurrent or refractory pancreatic adenocarcinoma	anti-CLDN 18.2 ADC	CMG901 injection	DLT, ORR
NCT05009966	I	China	–	Gastric/GEJ Pancreatic Solid tumors	CLDN 18.2-positive locally advanced or metastatic refractory solid tumors	anti-CLDN 18.2 ADC	SYSA1801 injection	DLT, MTD
NCT05525286	I/II	China	1st line	Gastric/GEJ Pancreatic	CLDN 18.2-positive, unresectable or metastatic pancreatic adenocarcinoma	anti-CLDN 18.2 ADC	SOT102 single agent SOT102 + nab-paclitaxel + gemcitabine	MTD, RP2D, ORR
NCT05980416	I	US	2nd/3rd line	Gastric/GEJ Pancreatic	CLDN 18.2-positive, unresectable pancreatic adenocarcinoma that failed prior therapy	anti-CLDN 18.2 ADC	EO-3021	DLT
NCT06038396	I/II	China	2nd/3rd line	Gastric/GEJ Pancreatic Solid tumors	CLDN18.2-positive, advanced or metastatic solid tumors that failed 1 line of therapy	anti-CLDN 18.2 ADC	RC118 + toripalimab	DLT, ORR, AE
NCT05934331	II	China	–	Gastric/GEJ Pancreatic	CLDN 18.2-positive, locally advanced GI cancers	anti-CLDN 18.2 ADC	LM-302 + toripalimab	PFS
NCT05365581	I/Ib	US, Japan, Korea	–	Gastric/GEJ Pancreatic	CLDN 18.2-positive, unresectable or metastatic pancreatic adenocarcinoma	bi-specific T-cell engager for CLDN 18.2 and CD3	ASP2138	DLT
NCT06005493	I/II	International	–	Gastric/GEJ Pancreatic Solid tumors	CLDN 18.2-positive locally advanced or metastatic solid tumors	bi-specific T-cell engager for CLDN 18.2 and CD3	AZD5863 (subcutaneous or IV)	DLT, MTD
NCT05482893	I	US	–	Gastric/GEJ Pancreatic	CLDN 18.2-positive, unresectable or metastatic pancreatic adenocarcinoma	bi-specific T-cell engager for CLDN 18.2 and CD47	PT886	DLT, MTD
NCT05583201	I	China	–	Gastric Pancreatic Solid tumors	CLDN 18.2-positive, advanced solid tumors	anti-NKG2DL/CLDN 18.2 CAR-T	KD-496	DLT
NCT05994001	I/II	China	2nd/3rd line	Biliary tract	CLDN 18.2-positive, unresectable biliary tract cancer that failed standard treatment	anti-CLDN 18.2 ADC	Cardonilimumab single agent LM-302 single agent	ORR
NCT05190575	II	China	2nd/3rd line	Biliary tract	CLDN 18.2-positive, unresectable biliary tract cancer that failed standard treatment	anti-CLDN 18.2 monoclonal antibody	TST001	ORR

GEJ, gastroesophageal junction; CAR-T, chimeric antigen-receptor T-cell; ADC, antibody drug conjugate; DLT, dose limiting toxicity; OS, overall survival; MTD, maxi-mum tolerated dose; PFS, progression free survival; DFS, disease free survival; RDE, recommended dose for expansion; RP2D, recommended phase 2 dose; ORR, overall response rate; AE, adverse events.

CLDN18.2 has potential as a diagnostic and prognostic tool as it is not expressed in normal pancreatic tissue but is aberrantly activated during malignant transformation. High levels of CLDN18.2 have been reported in PDAC and its precursor lesions, pancreatic intraepithelial neoplasms, and IPMN. A study analyzed 93 cases of primary PDAC for CLDN18.2 expression and found 88 (94.5%) cases showed positive expression (12, 28). Additionally, CLDN18.2 has a specificity and sensitivity of 93% and 79% in identifying primary PC in metastatic adenocarcinomas, giving more credence to its diagnostic role (19). As a prognostic indicator, CLDN18.2 is believed to be a poor indicator as it has been linked to stage III/IV PC, with higher expression in those with metastatic disease or lymph node invasion (13). While there is no proven direct correlation between CLDN18.2 and overall survival, the association with late stage and metastatic disease suggests a poorer prognosis in CLDN18.2-positive patients.

CLDN18.2 expression has also been reported in biliary tract malignancies, although less is known about its implications. It is thought to play a role in promoting tumor growth and metastasis (38). Similar to pancreatic and gastric cancer cells, CLDN18.2 in malignant biliary tract cells is regulated by the PKC pathway and DNA methylation at the promotor region, while increased CLDN18.2 promotes tumorigenesis by activating the EGFR and ERK1/2 pathway (38). *In vivo* studies have demonstrated that the suppression of CLDN18.2 significantly reduced cell growth and invasiveness of bile duct cancer cell lines (38).

One of the primary reasons of poor outcomes in biliary tract cancers is their late presentation and the difficulty in accurate diagnosis at early stages. There is increasing evidence that CLDN18.2 has potential as biomarker for the diagnosis of biliary tract cancers as it is only found in malignant biliary or gallbladder epithelium (116, 117). Multiple studies have shown a high rate of CLDN18.2 in both primary and metastatic biliary tract cancers, such as intrahepatic and extrahepatic cholangiocarcinoma (19, 116–118) CLDN18.2 was also frequently seen in premalignant lesions such as biliary intraepithelial neoplasia (BilIN), suggesting it can be a useful marker for patients undergoing serial monitoring for precursors of biliary tract cancers (113, 119).

### 4.9 Claudin 23

Finally, CLDN23, a non-classical CLDN, differs structurally from classical CLDNs with elongated cytoplasmic tails and altered extracellular loops. These distinctions impact its TJ formation and interactions with associated proteins, particularly evident in the precise localization of CLDN3 and CLDN4 at the TJ plasma membrane compared to the nonlinear localization of CLDN3 and CLDN4 in the absence of CLDN23 demonstrated by Raya-Sandino et al (39). Beyond its barrier role, CLDN23 is implicated in signaling pathways and organ-specific functions. Its tissue-specific expression pattern, primarily in the gastrointestinal tract, liver, and germinal center B cells, suggests specialized roles in these organs, potentially linked to oncogenic activity in intestinal-type germinal cells (39).

The clinical significance of CLDN23 varies across cancers. In gastric cancer, negative CLDN23 expression correlates with longer overall survival (120), while decreased CLDN23 expression in colorectal cancer are associated with shorter overall survival (121). In PC, CLDN23 may regulate invasion and metastasis, with decreased levels linked to factors promoting tumor invasion and metastasis (122). CLDN23’s dual roles in barrier function and signaling make it a promising biomarker and a target for further investigation in organ-specific malignancies, offering potential avenues for targeted therapies in the future (43).

## 5 Targeting claudins in pancreato-hepato-biliary malignancies

The distinctive expression patterns and function of CLDNs, particularly in certain cancers, highlights their potential as targets for anticancer therapies. Carcinogenesis often leads to the redistribution of CLDNs from their typical apical location to the entire membrane, affecting cell polarity and molecule localization (44, 57, 123). A true structural characterization has been limited for CLDNs in part due to research reagents and advances such as MBP-CC1 chimeric claudin have been developed to further augment future translational research (124). Kyuno et al. suggest that while mAb targeting claudins may not access certain areas in normal cells, in cancer cells, claudins are more accessible on the basolateral membrane, potentially allowing for direct therapeutic binding. (35).

However, the development of targeted therapies is complex, as no cancer-specific CLDNs have yet been identified, and the high homology among CLDNs poses a significant challenge with a risk of off-target effects (35). Additionally, the exact role of CLDN signaling in cancer progression is not fully understood, which may limit the effectiveness of some CLDN-targeted treatments.

**Figure 2:**
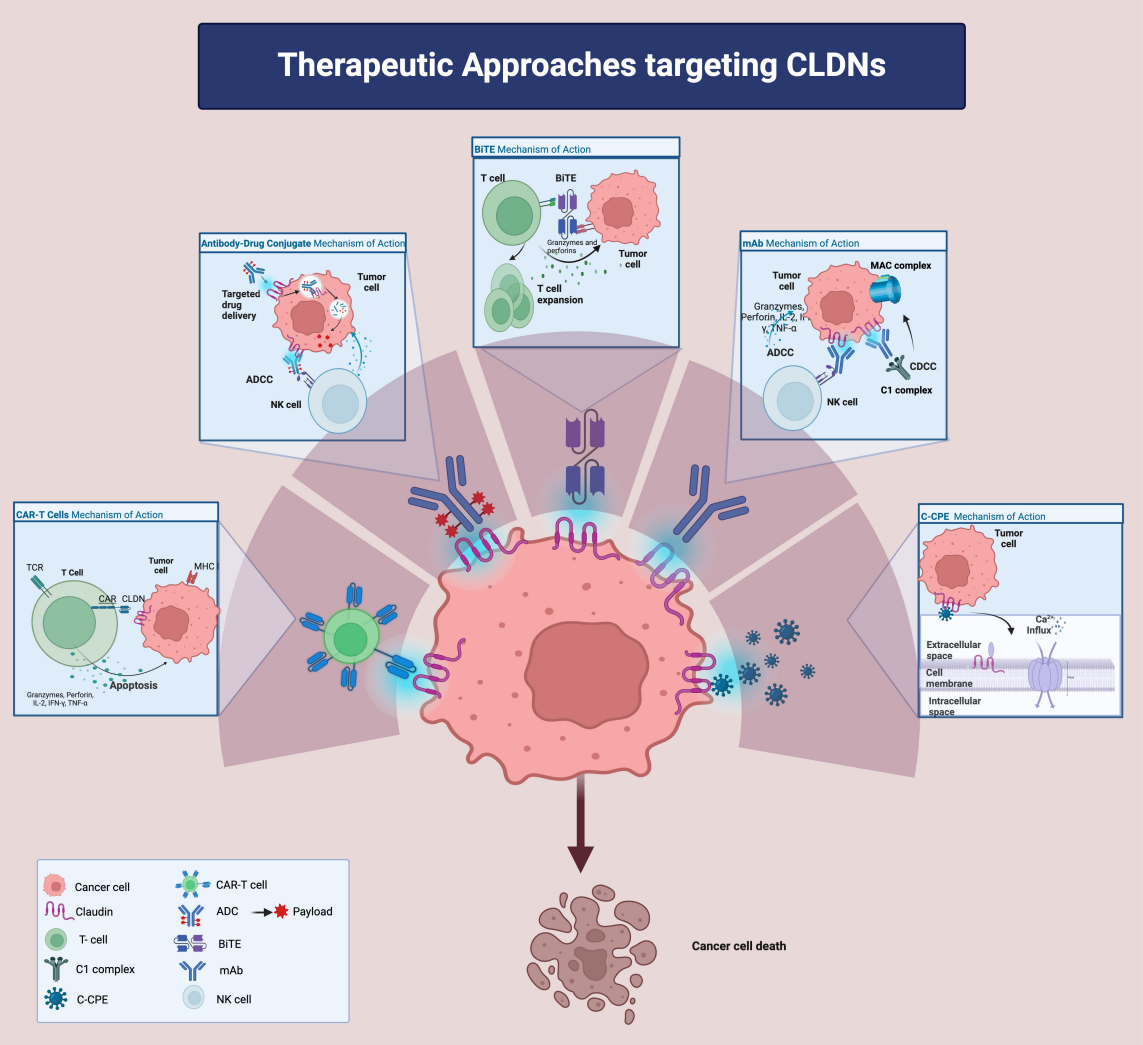
Therapeutic approaches targeting Claudins. ADC: antibody-drug conjugate; ADCC: antibody-dependent cellular cytotoxicity; CDCC: complement dependent cytotoxicity; CLDN: claudin; BiTE: bi-specific T cell engager; CAR: chimeric antigen receptor; IFN-g: interferon-g; IL-2: interleukin-2mAB: monoclonal antibody; MHC: major histocompatibility complex; TCR: T cell receptor; TNF-a: tumor necrotic factor-a;C-CPE: c terminus of clostridium perfringens enterotoxin; NK cell: natural killer cell.

Despite these challenges, several CLDN-targeted agents, including mAbs, CLDN-associated bacterial products, CLDN-directed mAb-drug conjugates, bispecific T cell engagers (BiTEs), and chimeric antigen receptor T-cells (CAR-T) (**Figure 2**), are under investigation in pre-clinical and clinical studies for various solid malignancies. Notably, zolbetuximab, a chimeric mAb targeting CLDN18.2-positive cancer cells, has demonstrated efficacy in clinical trials, especially in gastric and gastro-esophageal junction adenocarcinomas (20), and is now being evaluated in PC (125). Other promising agents including hu7v3-Fc (humanized heavy chain fused antibody) and TST001 (recombinant humanized mAb) (126–128), both targeting CLDN18.2, with unique properties such as higher affinity and faster tumor uptake that enhance their therapeutic potentials, are under development.

Other CLDN-directed mAbs have shown promise in pre-clinical models. Given the established heightened expression of CLDN4 in PC, it may represent a promising target for mAb in PC as *in vivo* studies in mice (69) have found that anti-CLDN4 mAbs can significantly reduce tumor growth in colorectal and gastric tumors and work synergistically in combination with chemotherapy in colorectal cancer (129).

In preclinical study of HCC, a humanized CLDN1-specific mAbs exhibited activity against the first extracellular loop of CLDN1 in a large series of patient-derived *ex vivo* model systems, *in vivo* CDX and PDX mouse model (47). Roehlen et al. demonstrates the CLDN1 mAb suppressed expression of EMT markers such as EPCAM and fibronectin 1 thus altering the tumor cell plasticity (47). Furthermore, treatment with CLDN1 mAb in HCC spheroids harbored increased gene expression of immune effector function, proliferation while immunosuppressive genes were downregulated (47), thus exerting immunostimulatory effects on the TME, offering potential treatment avenue of exploration in combination with immune-oncological approaches and multi-kinase inhibitors for advanced refractory HCC.

Ulteriorly, in consideration of the observed apoptosis of gallbladder cancer cells and inhibited cell invasion induced by downregulating CLDN1 expression (46), there is a strong rationale for considering CLDN1 as a novel target for the treatment of GBC with anti-CLDN1 mAbs.

Albeit progress has been lacking, bacterial products have also been explored as antitumor agents for their ability to target CLDNs, either by hijacking tissue-specific membrane proteins or by loosening cell junctions. *CPE*, for instance, attaches to the extracellular loops of specific claudins (CLDN3, 4, 6, 7, 8, 9, 14, and 19) (7). Considerable efforts were made to develop a *CPE*-based targeted cancer due to its pore forming (oncoleaking) ability and direct cytotoxic effect that is stronger against poorly differentiated PCs than the well differentiated ones (130, 131). Concomitantly the COOH- terminal receptor binding domain of *CPE* (C-CPE), without a known cytotoxic effect, is employed to facilitate drug delivery and susceptibility (132, 133). The CLDN4 binder C-CPE 194 for example is reported to enhance cytotoxicity of S-1 and gemcitabine on pancreatic cancer cell lines via increased MAPK pathway (134). Additionally, C-CPE based compounds such as C-CPE- fused PSIF (protein synthesis inhibitory factor derived from Pseudomonas Aeruginosa) (135, 136) or C-CPE-DTA (diphtheria toxin fragment A) (137) appear to be especially toxic to CLDN4 cancer cells *in vivo* and *in vitro*. The oncoleaking capability is also being exploited in the context of suicide gene therapy. A study by Pahle et al. employed translationally optimized *CPE* (optCPE) vectors using cell line-derived and patient-derived PC xenografts (138) leading to an increased apoptosis and necrosis signaling after implantation of the optCPE gene into PC cells. We await for further advancements in this aspect for CLDN targeted therapy development.

Antibody-drug conjugates (ADC) are a potent en vogue class of antitumor agents that enhance the efficacy of mAbs by carrying and delivering cytotoxic molecules directly to cancer cells. These agents have the capacity to circumvent the limitations of monoclonal antibody monotherapy. To date, more than twelve CLDN-ADCs have been created, with over a hundred ongoing trials in early phases of clinical research (28, 139, 140). Promising pre-clinical studies on SYSA-1801, a human mAb conjugated with the cytotoxic drug MMAE and directed at CLDN18.2, have led to its advancement into human clinical trials in advanced solid tumors (NCT05009966), including PC (28). Additionally, two phase I trials are investigating other CLDN18.2-targeting ADCs, namely CPO102 (NCT05043987) and CMG901 (NCT04805307), for treating various solid tumors such as pancreatic, gastric and gastroesophageal junction cancers.

BiTEs are an emerging CLDN-based therapeutic strategy for PCs (141), aiming to redirect the body’s T cells against cancer cells by linking the T-cell CD3 marker with the tumor antigen, in this case, CLDN18.2. This approach has shown promise in inhibiting pancreatic and gastric cancer growth *in vitro* with a favorable safety profile in *in-vivo* studies in mice (142). These findings have supported the development of bispecific and tri-specific monoclonal antibodies that enhance antibody-dependent cellular cytotoxicity while minimizing toxicity and are being currently tested in several phase I studies. The novel tetravalent bispecific platform, ABP-150, targeting CLDN18.2, has demonstrated significant antitumor activity with improved efficacy over the bispecific form in treating gastric cancer due to increased cytotoxicity *in vitro* and *in vivo* (143). Additionally, Q-1802, a humanized bispecific antibody targeting both CLDN18.2 and PD-L1, has undergone a phase I monotherapy dose-escalation study with subsequent dose-expansion study including 17 patients with advanced gastric adenocarcinoma, PC, and biliary tract cancer, showing promising safety and antitumor activity at doses up to 20mg/kg (128). The interim results presented at the Gastrointestinal Cancers Symposium in 2023 included that among the 9 subjects in the dose-expansion phase with CLDN18.2 positive expression with measurable lesions, two achieved a partial response and 4 achieved stable disease.

Cellular therapies, notably CAR-T treatments, have made significant strides in hematologic cancers but are still facing challenges in solid tumors due to the heterogeneity of the tumor-associated antigens and the suppressive TME. Interest in CAR-T therapy has grown with the discovery of their potential to target CLDNs such as CLDN6 and CLDN18.2, overexpressed in certain tumors. Engineered CAR-T cells have shown success in pre-clinical models, leading to sizeable tumor regression with minimal side effects (144).

In addition to CLDN18.2, the cancer-specific expression of CLDN6 has spurred increased interest in novel therapeutic strategies. This has led researchers to investigate the potential of CLDN6 as a target for precision medicine approaches (**Table 3**). Among these are the development of antibody-drug conjugates (43, 145) and bispecific antibodies (100). Additionally, CLDN6 is being explored as a target in CAR T-cell therapy for solid tumors (145, 146). Notably, promising results regarding disease control and manageable toxicity profiles have emerged from a phase I trial that employed CLDN6-specific CAR T cells in conjunction with an amplifying RNA vaccine (CARVac) (43, 139).

**Table 3:** Claudin 6-targeted Ongoing Clinical Trials.

Trial Name	Phase	Country	Treatment Setting	Cancer type	Patient	Therapeutic agent	Treatment arms	Primary Endpoint	Status
NCT05262530	I/II	International	2nd/3rd	Solid tumors	CLDN6-positive advanced or metastatic solid tumors that failed standard therapy	bi-specific T-cell engager for CLDN 6 and CD3	BNT142	DLT, ORR	Recruiting
NCT05317078	I	International	–	Solid tumors	CLDN6-positive locally advanced or metastatic solid tumors	bi-specific T-cell engager for CLDN 6 and CD3	AMG794	DLT	Recruiting
NCT04503278	I/II	International	–	Solid tumors	CLDN6-positive unresectable or metastatic solid tumors	RNA vaccine- augmented CAR-T	CLDN6 CAR-T/CLDN6 CAR-T(A) CLDN6 unmodified RNA-LPX/CLDN6 modified RNA-LPX	DLT	Recruiting

DLT, dose limiting toxicity; ORR, overall response rate.

In this review, we have described various CLDNs of potential therapeutic value in hepato-pancreato-biliary cancers. Further investigation is warranted to unravel the intricate regulatory mechanisms underlying CLDN expression and function in cancer. A more comprehensive understanding of the interplay between CLDNs and other cellular signaling pathways could pave the way for novel therapeutic targets and strategies. Additionally, overcoming the challenges posed by the high homology among CLDN family members is crucial for developing more effective and specific therapeutic agents with minimal off-target toxicity.

## 6 Conclusion

Claudins, with their pivotal role in cancer pathophysiology, hold promise as unique targets for therapeutic intervention. Their distinct expression patterns in cancer cells provide a foundation for developing innovative treatments. The field of claudin-targeted therapy, particularly in hepatobiliary and pancreatic cancers, though still in its nascent stages, is advancing rapidly. While some treatments have shown promising clinical efficacy, continued research is imperative to diversify and broaden therapeutic strategies, aiming to improve patient outcomes for these notoriously difficult-to-treat malignancies. The next steps in drug development should encompass further exploration of therapeutic approaches, including combinatorial strategies with chemotherapy and immune-modulating agents, as well as expanding their application to earlier disease stages where they could have a more profound impact.

## Footnotes

1
https://www.proteinatlas.org/
